# Supplementary material for: Are carbon emissions trading and green financial instruments synergistic? -Comprehensive quantitative research based on content analysis
Source: PLoS One. 2024 Mar 7;19(3):e0298601. doi: 10.1371/journal.pone.0298601 (PMC10919601; doi:10.1371/journal.pone.0298601)
Supplement: S1 Data — (ZIP) [file pone.0298601.s001.zip › Data/地级市Z_F工作报告.zip!/┴╔─■/┼╠╜⌡╩╨/└┤╫╘╣1⁄2╓┌║┼í╛┬φ┐╦╩2╛▌═°í┐_2015.docx]

各位代表：

　　现在，我代表市政府，向大会报告工作，请予审议，并请各位政协委员和其他列席同志提出意见。

　　一、2014年工作回顾

　　过去的一年，是盘锦经济社会发展面临挑战极为严峻的一年，也是我们经受考验，攻坚克难，拼搏奋进的一年。

　　面对经济下行的巨大压力，在省委、省政府和市委的正确领导下，坚持一张蓝图绘到底，紧紧抓住中央支持东北振兴重大历史机遇，统筹稳增长、调结构、促改革、惠民生，迎难而上，开拓创新，推动经济社会发展在逆势中向前迈出了坚实一步。

　　经济运行总体平稳。预计地区生产总值完成1426亿元，同比增长7%；固定资产投资1171亿元，增长3%；社会消费品零售总额增长12%。新 常态下，各项指标增速虽有所放缓，但依然稳步增长，高于全省平均水平，主要指标走在全省前列。这是在全国全省各地经济增速普遍下降、下行压力持续加大情况 下实现的全面增长，来之不易。粮食产量达到115万吨，大旱之年再创历史新高。社会就业更加充分，新增实名制就业7.1万人，城镇登记失业率控制在 2.9%以内，低于全省平均水平。市场物价保持稳定，居民消费价格涨幅控制在1.4%以内。

　　发展质量和效益不断提升。公共财政预算收入151.2亿元，增长1.6%，收入质量进一步提高。万元生产总值能耗下降3%，化学需氧量下降5%，二氧化硫排放量下降10%。工业用电量、货物周转量等实物量指标与经济同步增长，相互匹配，互为支撑。

　　结构调整取得积极进展。投资结构持续优化，工业、现代服务业项目投资比重达到90.3%；按新口径统计，实际利用外资4.8亿美元，其中工业利 用外资占比达到67.9%。科技创新能力增强，以盘锦大连理工大学为支撑的产学研协同创新体系不断完善，大学科技园挂牌运行；建成2个国家级重点实验室、 17个省级研发中心，引进建设了一批高端制造等高新技术项目，高新技术产品增加值增长20%。服务业增加值占GDP比重进一步提高。民营经济活力不断增 强，增加值占经济总量比重达到54.4%。

　　民生福祉持续改善。城镇常住居民人均可支配收入31083元，增长10%；农村常住居民人均可支配收入12860元，增长12%，城乡居民收入水平保持全省前列。财政用于民生领域支出达到135.4亿元，占总支出的66.7%。发展成果更多惠及人民群众。

　　过去的一年，困难超出预想，成果好于预期。经济社会发展既有量的扩张，更有质的提升，为今后发展奠定了坚实基础。一年来，我们主要做了以下几个方面工作：

　　（一）抓住用好重大历史机遇，转型发展开创崭新局面。去年，党中央、国务院作出新一轮支持东北振兴的重大战略部署。我们逐条逐项狠抓落实，争取 到国家湿地生态效益补偿试点、首批生态文明先行示范区等多项政策及重大项目，获得国家和省各类资金支持95.5亿元。城投债券发行得到国家大力支持，获批 额度位居全省第二。加强与大央企、大民企的对接合作，签署合作项目32个，总投资超过1500亿元。

　　倍受瞩目的兵器集团华锦石化改扩建项目及30万吨原油码头配套工程被国务院纳入政策支持范围。

　　（二）工作重心向沿海倾斜，辽东湾新区建设全面提速。立足发挥新区龙头带动作用，形成了举全市之力建设辽东湾的工作格局，推动新区在新的起点上加快发展。

　　持续推进港口建设。10个5万吨级以上泊位主体完工，港口吞吐量突破3000万吨,晋升为区域性重要港口。疏港铁路、疏港高速开通运行，沈盘铁 路加速铺轨，沈西工业大道盘锦出海通道启动实施。盘锦港起步工程通过国家环评验收，国家一类口岸即将获得批复，沈铁港前物流基地投入使用，保税仓、出口监 管仓封关运营，综合保税物流中心成功获批，为建设亿吨大港构筑了坚实支撑。

　　加快壮大产业规模。长春石化、瑞德化工等项目竣工投产，忠旺铝材、联成化学、宝来石化、振奥橡胶、汇福粮油等项目加快建设，亿元以上项目竣工19项，在建62项，完成投资181亿元。现代物流业快速发展。电子商务产业园顺利推进，大宗商品现货交易平台上线运营。

　　全面推进水城建设。城市规划展示馆投入使用，图书馆、科技馆等主体完工，基础设施工程全面展开，公交客运体系加快建设，城市功能不断完善。金帛海岸、江南风情园投入运营，兴隆七彩城、二界沟新渔港新渔村等加快推进。教育、卫生等社会事业和社会保障全面进步。

　　如今的辽东湾新区，规划布局更加科学合理，港产城联动发展、快速发展。一座现代化、生态宜居的港口城市正在崛起。

　　（三）千方百计推进项目建设，支撑发展能力显著增强。全市实施亿元以上项目370项,完成投资760亿元。这些大项目成为支撑转型发展的核心力量。

　　分类推进在建项目。将存量项目划分为ABCD四类加以推进，有效提高项目建设质量和速度。质量较高的A类B类项目比重达到94%。戴纳索丁苯橡胶、万达广场、丰源热力、台湾士林不夜城等82个投资超10亿元的重大项目加快建设，17个重大项目竣工投产。

　　实施常态化、专业化、定向定点招商。面向长三角、珠三角、京津冀、台湾等重点地区，高强度开展点对点招商，长效推进“市长洽谈日”请进来活动，台湾正新轮胎、青岛软控、中建材、中储粮等141个重大项目深入推进。

　　加快出口基地建设。制定实施一系列政策措施，培育壮大了2个省级出口基地、4个市级出口基地，支持一批出口型龙头企业加快发展。地产品出口增长41.9%。

　　全力保障要素供给。新增贷款97.4亿元，非信贷融资165亿元。落实新增建设用地指标8平方公里，调整建设用地规划9.5平方公里，集约节约用地水平明显提高，保障了重点项目需求。

　　（四）持续推进结构优化升级，产业实力竞争力不断提升。积极发挥科技支撑引领作用，组建精细化工、能源及海洋装备2个产业创新联盟，24个省以上重大科技项目加快推进。有效承接中关村高新技术产业转移，创新驱动能力明显增强。

　　推进工业提质增效，40个重大结构调整项目优质竣工，规模以上企业发展到525户，四个省级产业集群销售收入突破2000亿元。成功举办国际石 油与天然气装备博览会。在油价下行、外需乏力的不利形势下，辽河油田、华锦集团、辽河石化等大企业平稳运行，为全市经济稳定增长做出了突出贡献。

　　大力开展旅游季活动，组织30余项精彩纷呈的主题活动，通过旅游专列、高铁冠名“红海滩号”等举措扩大宣传，加强景区建设，完善服务功能，湿地旅游持续火爆。全年旅游企业营业收入增长11%。

　　启动服务业发展四年行动计划。省级服务业集聚区发展到6个，辽东湾临港物流园被评为国家级优秀园区。大洼专业市场集聚区、宏安国际物流中心、蓝 碧源五金汽配城等市场群加快建设，金社裕农连锁超市全面铺开，盘锦成为国家农产品流通体系和农村市场体系建设试点市。辽河文化产业园树起崭新形象。工业企 业生产性服务业分立取得明显进展。电子商务快速兴起，夜经济、楼宇经济加快发展。

　　采取修订基准地价、规范土地出让、举办房交会等举措，调整房地产结构，拉动市场销售。房地产投资增长25.7%，商品房销售额增长3.3%，均保持全省前列。

　　稳步推进农业产业化发展，一批知名农事企业相继入驻。设施农业快速发展，新增棚菜面积2.4万亩。盘山县成为国家现代农业示范区改革建设试点县，大洼县成为国家水稻高产创建整体推进县。

　　推出惠农贷款等金融产品支持农户生产经营。加强土地规范流转，积极培育农民合作社、家庭农场等新型经营主体。水稻生产综合机械化率达到88%， 走在全省前列。河蟹养殖面积、产量稳居全国地级市之首，大米河蟹北京展销会圆满成功，品牌价值进一步提升。农业经济呈现出多业态、多种模式竞相发展的良好 局面。

　　实施大规模调水工程，推进了双台子河闸除险加固、辽河口大型泵站改造等水利工程。这些工程为夺得农业丰收起到了重要作用。

　　（五）以农村环境综合治理为突破口推进城乡一体化，城乡面貌发生巨大变化。按照市委的决策部署，在全市395个行政村全面实施了造林绿化、道路 建设等九大类农村环境整治工程，推进了每村建设一个标准化超市、一个卫生室等“四个一”工程，同步发展镇村特色产业，推动教育、文化等公共服务均等化。我 们用一年时间完成了省部署的5年环境整治任务，赢得了省委、省政府的高度肯定和全市人民的广泛拥护，盘锦成为全省宜居乡村建设试点市。特别是经过深入实 践，找到了一条符合中央要求、切合盘锦实际的新型城镇化、城乡一体化发展道路，农村环境、社会关系以及农民思想观念、生产生活方式都发生了深刻变化。

　　加强城市建设管理。科学编制市域总体规划，高标准推进城市总体规划修编。建成盘锦北互通立交、辽河生态示范路一期等19项交通工程和20项市政工程，改造老旧小区84个。推进城市精细化、网格化、智能化管理，盘锦在创建全国文明城市测评中，连续三年位列全省第一。

　　狠抓生态环境建设。加大湿地保护，湿地生态效益补偿项目全面启动，完成湿地围栏等工程建设。大力实施碧水工程，辽河流域盘锦段水质进一步改善。继续实施蓝天工程，集中供热水平走在全省前列，空气环境质量不断提升。

　　（六）大力推进小微企业发展，全民创业创新进入崭新阶段。启动实施小微企业发展五年行动计划，全市小微企业数量增长13.3%，其中新登记公司制企业增长87%。

　　推进工商登记制度改革，全力降低投资准入门槛，放开审批限制。最大限度减轻税费负担，累计减免涉企各类税费1.1亿元，小微企业受益面不断扩大。

　　加强融资服务。民生银行入驻盘锦，小额贷款公司、融资担保机构分别发展到30家和37家。积极为符合条件的创业主体提供融资担保和贴息支持，妥善解决了创业者无抵押物贷款难问题。建立创业孵化基地、综合服务平台12个，为小微企业发展创造了良好环境。

　　（七）全面深化改革，发展动力活力明显增强。大力简政放权，取消或调整市县镇三级行政审批事项1332项。在全省率先公布了三级审批目录、搭建 了网上审批平台，全面推行并联审批、直联审批，将建设工程项目审批时限压缩到25个工作日，比省政府规定时限缩短15个工作日，事中事后监管服务不断加 强。

　　深化政府机构改革。完成了卫生、计生等8个部门整合，调整了市本级工商、质监、食品药品管理体制，实现了县区市场监管体系“三合一”建设。大幅精简政府部门内设机构和人员编制，重构内部组织形态。扎实推进事业单位分类改革，精简事业单位44家。

　　深化财税体制改革，推行财政零基预算管理，实现各级政府和部门预决算公开、“三公”经费公开。建立借用管还相统一的政府债务管理机制，有效防范了债务风险。

　　农村综合改革和价格体制等改革有序推进。加快户籍制度改革，盘锦成为全省落户门槛最低的城市。

　　（八）切实保障和改善民生，各项社会事业全面进步。始终把保障和改善民生作为政府工作的重中之重，年初确定的34件民生实事全面完成。

　　建立城乡统一的居民基本养老保险制度，基础养老金月人均提高20%。城镇企业退休职工养老金月人均增加165元。在全省率先开展居民医疗保险城乡并轨工作，辽东湾新区试点运行良好。完善城乡居民大病保险制度，城乡医保年补助标准人均提高40元。

　　居民低保标准提高10%，其中县域低保标准全省第一。实现了1.1万名农村贫困人口脱贫。新建扩建了一批社会福利院、老人日间照料中心、农村互助幸福院。改造城市棚户区16652户、农垦危房9310户。解决了历史遗留的2516户居民房屋产权证办理问题。

　　市中心医院荣获“中国医疗机构公信力示范单位”，进入全国三甲医院百强行列。启动建设市精神疾病专科医院。两县公立医院改革深入推进，26个镇 级卫生院实现公益性回归，国家基本药物制度继续向村级延伸。组建了市医患纠纷调解中心。全面实施单独两孩政策。实施出生缺陷干预工程，人口质量不断提高。

　　各位代表，教育公平是社会公平的重要基础，是人民群众的迫切期待。我们全面加强教育资源均衡配置，坚持义务教育划片招生、就近入学，严把各道关 口，彻底杜绝非正常流动，建成兴隆中学、新世纪小学、光正实验学校，使多年来社会反映强烈的择校问题、大班型问题得到了妥善解决。盘山县成为全国义务教育 发展基本均衡县。全面推行学校食堂食材统一配送，切实保障了校园及周边食品安全，为孩子们创造了公平、健康的成长环境。

　　文化体育事业加快发展。全国第十二届美展港澳台作品展圆满成功，兴隆台区跻身全国文化工作先进县区行列。成功举办省第八届少数民族运动会和中超联赛辽宁主场赛事，获得第十三届省运会承办权。

　　大伙房水库输水配套工程优质竣工，城区、县城、辽东湾新区及部分镇45万居民吃上了安全洁净的放心水，结束了饮用地下水的历史。解决了农村6万 人饮水安全问题。城乡公交客运一体化建设走在全省前列。规范和加强公共服务，与群众生活密切相关的供电、通信等行业服务水平进一步提高。

　　坚持每月开展一次安全稳定调度评估，狠抓各类隐患排查治理，实现了安全生产监管、社会安全管理、信访稳定的常态化、法治化。重拳整治油气管线占 压、违法排污、黑出租车、食品药品等安全隐患。积极化解社会矛盾，信访目标考核继续保持全省第一。严厉打击黑恶势力等各类违法犯罪行为，完善视频监控系 统，初步构建起全民反恐的工作格局，群众安全感明显提升，社会更加和谐稳定。

　　一年来，我们深入开展党的群众路线教育实践活动，聚焦“四风”问题，狠抓作风建设，大力实施提高机关工作效率等专项行动，促进了各项工作的有效 落实，解决了一批事关群众切身利益的实际问题。全市上下服务群众、推动科学发展的意识和能力不断提高，为民务实清廉的政府形象进一步树立。

　　自觉接受市人大及其常委会的法律监督、工作监督和市政协的民主监督，认真办理人大代表建议和政协提案，办复率均达到100%。

　　坚持依法行政，出台《市政府重大行政决策规则》，把公众参与、专家论证、风险评估等确定为重大行政决策的必经程序，严格规范执法行为，法治政府建设扎实推进。

　　完善《市政府工作规则》，制定实施一系列配套管理制度，使政府工作走上了制度化、科学化的轨道。

　　加大行政监察和审计监督力度，强化政务督查、通报、问责，推进政务公开，勤政廉政建设有效加强。严格执行中央“八项规定”，全市“三公”经费同比减少20.7%。

　　国防动员、双拥共建深入开展。妇女儿童、老龄慈善、外事侨务、民族宗教、防震减灾、爱国卫生、人民防空、广播电视、红十字会、住房公积金和残疾人、地方志、统计、气象等工作都取得了长足进步。

　　各位代表，2014年是盘锦建市30周年。30年来，历届市委、市政府团结带领全市人民，艰苦创业，奋力拼搏，夺取了一个又一个重大胜利，用智 慧、勇气和汗水书写了恢宏壮阔的历史篇章。全市经济总量比建市之初翻了近7番，城乡居民收入分别增长28倍和29倍。向海发展的空间格局全面拉开，以港强 市的战略地位成功确立，多元产业体系形成支撑，群众生活更加富庶幸福，一个更具实力活力和竞争力的滨海新盘锦正以崭新的面貌走向全国、走向世界。

　　盘锦今天的发展局面，来之不易。这是省委、省政府和市委正确领导的结果，是市人大及其常委会、市政协有效监督的结果，是全市人民团结奋斗的结 果。在此，我代表市政府，向全市人民，向人大代表、政协委员，向各民主党派、工商联、无党派人士和人民团体，向驻盘部队广大官兵，向辽河油田、华锦集团、 辽河石化、长城钻探、盘锦港务集团等大企业，向所有关心支持盘锦发展的各界朋友，表示衷心的感谢！

　　各位代表，当前盘锦同全国、全省一样，经济发展进入了增长速度的换挡期，地区生产总值、财政收入、投资等指标增速均有所放缓。这是新常态下，宏观大环境等因素影响的结果，经济规律作用的结果。

　　同时，全市经济社会发展中还存在着一些矛盾和问题：经济结构仍需深度调整优化，服务业占比较低，战略性新兴产业尚未形成支撑，科技创新能力依然 不足；受市场等因素影响，原油和部分石化产品价格下降，一些企业经济效益下滑；财政收支矛盾突出，各级财税增收压力加大；改善民生的任务依然繁重，等等。 对此，我们一定高度重视，采取有力措施，认真加以解决。

　　二、2015年工作安排

　　今年是“十二五”规划的收官之年，全面深化改革的关键之年，也是全面推进依法治市的开局之年。做好今年政府工作，意义重大。

　　综合分析形势，今年我市面临的外部环境依然复杂多变，但总体向好的基本面不会改变。

一是，党中央、国务院和省委、省政府为适应新常态、引领新常态，把稳增长摆在更加重要的位置，推出一系列重大发展战略和政策举措，必将形成新的增长动力，市场投资能力和投资需求将得到加强。

　　二是，从我市拉动增长的“三驾马车”来看，都有积极变化，特别是项目投资势头强劲，形成核心拉动力，投资对经济增长的贡献率达到80%左右。随 着政府投资的下降，市场化投资已成为关键拉动力量。通过招商引资的积累，我们引进实施了300多个产业项目，今年预计投资将超过770亿元，为稳增长、调 结构、促转型提供重要支撑。同时随着旅游、电子商务等消费热点的培育，出口基地建设的加快推进，以投资为核心，消费、出口共同发力，经济增长的动力将更加 强劲。

　　三是，近年来我们立足打基础、谋长远，通过适度举债，建设了一大批基础设施工程，形成了以辽东湾新区为龙头全面向海发展的开放格局，创造了港 口、区位、空间等多位一体的综合优势。这些优势正在有序释放，成为我们新常态下区域竞争的最大优势，成为我们抢占发展制高点的重要基础。只要我们抓住机 遇、勇于担当，主动作为、持续发力，今年经济社会发展的形势就一定会好于去年，盘锦就一定能够在新一轮东北振兴中率先突破。

　　我们一定深刻理解并用好新常态下盘锦的新机遇、新优势，牢牢抓住发展这个第一要务不动摇，推动各项事业行稳致远，不断开创盘锦经济社会发展新局面。

　　今年政府工作的总体要求是：全面贯彻党的十八大和十八届三中、四中全会精神和习近平总书记系列重要讲话精神，贯彻落实市委六届八次全会决策部 署，坚持“一条主线、三大任务、四个转型”的总体布局，主动适应经济发展新常态，自觉顺应百姓生活新期待，有序释放向海发展新优势，把转方式调结构促转型 放在更加重要位置，狠抓改革攻坚、突出创新驱动、持续扩大开放、加强民生保障，有效发挥投资的关键作用，着力培育形成全面转型的新动力、新支撑，努力实现 有质量的速度、有深度的改革、有福祉的发展，全面建成小康社会，奋力开启向海发展、全面转型、以港强市新航程。

　　综合考虑各方面因素，今年我市经济社会发展主要预期目标是：地区生产总值增长7%；规模以上工业增加值增长7%；固定资产投资增长8%；公共财 政预算收入增长3%；社会消费品零售总额增长12%；城镇常住居民人均可支配收入增长10%；农村常住居民人均可支配收入增长11%。实际工作中，我们将 努力推动主要指标增速高于全省平均水平，继续走在全省前列，做到调速不减势、量增质更优，实现科学发展、可持续发展、包容性发展。

　　各位代表，到今年年底，盘锦要全面建成小康社会，比全国全省总体目标提前5年实现，这是市第六次党代会作出的决策部署。我们要建设的小康，不仅 是指标上的小康，还要是生活感受上的小康；不仅在城市里实现小康，还要在乡村中实现小康；不仅在人均上实现小康，还要在个体上实现小康。我们一定全力以 赴，集中攻坚，奋力实现这一宏伟目标，加快建成一个让全市人民普遍感到幸福、引以自豪、真正的小康盘锦。

　　围绕实现今年任务目标，我们将切实把工作着力点放到转方式调结构促转型上来，依靠市场的力量、创新的力量，在推进新型工业化、信息化、城镇化和农业现代化中挖掘培育新的增长点，加快发展新技术、新产业、新业态、新模式，以发展保障改善民生。重点做好十个方面工作：

　　（一）全面落实新一轮东北振兴重大战略决策。集中力量推进兵器集团华锦石化改扩建及30万吨原油码头工程开工建设。抓好40个投资超10亿元的重大存量产业项目，加快推进北沥润滑油加氢、重汽LNG装备生产改造基地等30个重大合作项目。

　　积极对接上级部门，全力抓好湿地生态效益补偿、资源型城市可持续发展试点等重大政策争取工作，努力纳入国家和省政策支持范围，培育新的竞争优势。

　　实现盘锦全面转型发展，必须以开放为牵引。我们要实施更加积极的开放战略，主动对接国家“一带一路”、京津冀协同发展等战略平台，积极参与中蒙俄经济走廊、中韩等自贸区建设，打造辽宁联结欧亚的重要出海通道，在更大范围、更宽领域、更深层次上融入全国全省开放大格局。

　　借鉴上海自贸区模式，以贸易便利化、投资便利化为主攻方向，加快形成更加有利于开放的营商环境，着力构建支撑全面开放的经济新体制。

　　大力发展口岸经济。加快保税物流中心建设，积极申报保税物流园区、综合保税区，实施关检通关合作，确保3月底前晋升为国家一类口岸，成为国家粮 食进出口指定口岸。有序释放综合优势，推进港口功能向陆域腹地延伸、向海外拓展，发展出口加工、国际贸易、仓储物流等产业，以港口和口岸拉动全市经济发 展。

　　加大出口基地建设力度。推动更多的盘锦产品、技术、装备走向国际市场，确保地产品出口8亿美元，力争两年内建成2个国家级出口基地、5个省级出口基地。鼓励有条件的企业“走出去”,开展对外投资，参与国际合作。

　　牢固树立开放意识。我们要自觉地把工作着力点聚焦到开放上来，以开放的思维、开放的胸怀谋划工作，用开放的举措推动发展，全面提高开放水平。

　　（二）开创辽东湾新区建设新局面。今年是新区建设10周年，是新区发展的重要时间节点。我们要进一步凝聚形成举全市之力建设新区的强大合力，要用当年建设兴隆台区的气魄和干劲来开发建设辽东湾，实现新区发展新跨越。

　　完善提升城市功能。以承办第十三届省运会为契机，全面推进路水电暖气等70项公建工程，启动建设运动员村，健全教育、文化、客运公交等公共服务，完善房地产、旅游、商贸等功能配套，建设现代化生态宜居新港城。

　　加快推进港口建设。新建续建9个5万吨级以上泊位，开工建设15万吨码头和航道工程，规划建设10万吨级粮食码头等专业化深水泊位，整合作业区资源，港口吞吐能力达到5000万吨。完善集疏运体系，开工建设阜盘铁路，实现沈盘铁路、沈西工业大道盘锦出海通道竣工通车。

　　发展壮大主导产业。支持长春石化、和运集团等重点企业加快发展，促进宝来石化、瑞德化工等新装置稳产运行，推进忠旺铝材、联成化学、汇福粮油等项目加快建设，启动建设中储粮等项目，着力引进一批重大项目、高新技术项目，壮大石化及精细化工、装备制造等产业规模。

　　大力创新体制机制。率先复制上海自贸区模式，探索建立新的投资和服务贸易管理体制。启动建设金融中心，加快金融机构集聚和融资平台建设。全面加 强与国内外发达地区的交流合作，借助周边城市的资源发展新区，把新区加快建设成为高端要素的集聚区、辽宁沿海开发开放的示范区。

　　创建全省乃至全国有影响力的创业之城。推进创业孵化基地、政策体系、服务体系建设，以优质的生活环境、市场环境、政务环境，广泛吸引各地区、各行业创业者来新区投资兴业，打造辽宁创新创业的新高地。

　　各位代表，辽东湾新区承载着盘锦的未来。我们坚信，经过全市上下的不懈努力，一个全新的港口新城一定会巍然屹立在渤海之滨。

　　（三）全面深化各项改革。落实市委决策部署，以处理好政府与市场关系为核心，着力深化具有支撑作用的关键改革，牵引和带动其他领域改革，不断把改革红利转化为发展新动能。

　　深化经济体制改革。稳步推进市粮库、供水企业、公用事业等国资国企改革重组。扎实推进王家农场改革试点，通过规模化生产、集约化经营，实现国有农场集团化发展。深化国有苇场体制改革，推进苇场公司化经营，建立现代企业制度，实现资源资产资本的有效整合。

　　深化财政体制改革。建立全面规范、公开透明的预算制度。实施零基预算管理，硬化预算约束，今后一律不再追加部门支出预算，因不可抗力和重大政策 调整确需追加的，严格按照规定程序报批。健全专项资金管理制度，所有支出均由集体讨论决定。完善借用管还相统一的政府债务管理制度。创新政府投融资机制， 积极推行政府和社会资本合作（PPP）等多种投融资模式，激活社会投资，多渠道筹措建设资金。

　　深化行政体制改革。大力简政放权，完善权力清单，制定责任清单和负面清单，加强网上审批平台建设，实现50%以上行政审批项目网上审批。全面实施营业执照、组织机构代码证和税务登记证“三证合一”制度。加快事业单位、镇级机构改革步伐。完成公务用车改革。

　　深化城乡一体化改革。抓好户籍、社保等配套改革，探索城乡统筹发展的新路子。开展规划“多规合一”试点。加快推进自然保护区、城乡生态环境保护管理体制改革。

　　（四）强力推进招商选资和项目建设。越是形势严峻，越要主动作为，以更大力度抓招商、抓项目。确保亿元以上项目引进150个，新开工100个，续建232个。

　　分类推进重点项目建设。建立定期分析和统筹协调推进机制，启用实时调度跟踪预警系统，加强动态考核，努力提高项目履约率、开工率和竣工率。

　　加大招商选资力度。面向京津冀、长三角、珠三角、港澳台等重点地区，高密度走出去，坚定不移地抓好“市长洽谈日”请进来活动，全力引进一批科技含量高、竞争力强的重大项目。建立项目跟踪推进和考核机制，确保招商取得实效。

　　增强要素保障能力。创新融资方式，提高运用资本市场的能力和水平，抓好信贷投放、债券发行、企业上市等多渠道融资。严格依法管理土地，提高土地节约集约利用水平，保障发展用地需求。

　　（五）加快产业结构优化升级。强化创新驱动，用全面创新推动三次产业发展更加协调、结构更加优化。

　　围绕建设世界级石化及精细化工产业基地、国内领先的能源装备制造基地，大力推进工业提质增效。主动帮助企业解决实际问题，重点支持辽河油田、华锦集团、辽河石化等大企业加快发展，稳定经济增长。

　　提高工业自动化水平。加快智能装备和先进自动化设备的推广应用，鼓励引导规模以上企业率先升级改造，提高生产效率和市场竞争力。

　　深入实施创新驱动战略。主动承接中关村等高新技术产业转移，打造以盘锦大连理工大学、曙光计算机为龙头的辽东湾高新技术产业园区，以中蓝电子为 龙头的兴隆台科技产业园。建设一批高水平的协同创新平台和研发转化基地，抓好陆海研究院、忠旺实验室、和运研究院等研发机构发展，加快科技孵化器建设，提 高科技成果转化率。围绕产业链部署创新链，集中科技专项资金，支持一批重大科技项目，支持专利发明和关键技术攻关，增强自主创新能力。探索设立新兴产业创 业投资引导基金，推动创业创新和产业升级，发展新材料、海洋工程装备、通用航空、电子信息等新兴产业。

　　大力实施服务业四年行动计划。提升服务业集聚区集聚效应，6个省级服务业集聚区营业收入增长20%以上。

　　推动商品市场与电子商务、现代物流融合发展，加快大洼专业市场集聚区等市场群建设，做大做强辽东湾新区、双台子区电商产业园，完善现代流通市场体系。支持金社裕农公司深化改革，拓展经营网络。大力培育夜经济、总部经济，形成新的增长点。

　　调整优化房地产业结构。大力推进棚户区改造。坚持市场导向，积极发展旅游地产、文化地产、养老地产等特色地产。继续办好房交会，支持油田外围职工回迁，扩大市场销售，促进房地产业平稳健康发展。

　　加快转变农业发展方式，走产出高效、产品安全、资源节约、环境友好的现代农业发展道路。深入开展国家现代农业示范区改革建设试点工作，加强水稻现代化示范区建设，提高生产技术装备水平，确保粮食丰产丰收。抓好24个重点项目建设，促进农业产业化发展。

　　大力推进农业结构调整。发展壮大大米、河蟹两大产业，继续办好大米河蟹北京展销会。加快发展设施农业，新建设施农业小区30个。把泥鳅鱼产业作为重要的富民产业来抓，构建孵化、养殖、加工、出口于一体的产业链，打造全国泥鳅鱼产业第一市。

　　创新农业经营体系。规范和加强土地流转，培育种养大户、家庭农场、农民合作社、农业企业等新型经营主体，积极发展多种形式的适度规模经营，让农民从产业链增值中获取更多利益。

　　加强农业综合生产能力建设。提高技术服务、职业培训、金融服务等保障水平。加快建设双台子河闸除险加固、辽东湾海堤、大辽河综合治理、绕阳河蓄水等水利工程，提高防灾减灾能力。

　　（六）大力发展生态旅游业。转变旅游发展理念，把发展旅游业与扩大消费、调整结构、促进创业结合起来，拉长旅游链条，形成以旅游业为龙头，带动住宿、餐饮、购物、娱乐等行业快速发展的良好局面，创建国家级生态旅游示范区，打造国际重要的湿地休闲旅游目的地。

　　加快重点景区建设。抓好辽东湾旅游度假区、红海滩旅游度假区、辽河口湿地旅游度假区等重点区域建设，打通海上通道，把旅游资源整合起来，提高开发利用水平。

　　支持金帛海岸、绕阳湾、疙瘩楼度假区等项目加快建设，确保台湾士林不夜城、黄金珠宝城、天沐温泉、二界沟新渔港新渔村等项目建成运营，促进蓝天城体验中心、多乐高娱乐城等项目落地开工，树起旅游业的新形象。

　　加强旅游基础设施建设。完善景区道路、停车场以及水、电等基础设施，加快信息咨询、餐饮、购物等配套设施建设，提高农家乐、渔家乐等标准化水平，为游客提供更加便捷舒适的旅游服务。

　　丰富旅游产品和文化内涵。推动旅游与文化深度融合，保护和发掘辽河口文化等特色文化，加快广厦艺术街等项目建设运营，把辽河文化产业园打造成为 北方最大、享誉全国的创意与设计服务产业基地。通过组织体育等各种赛事活动扩大影响。围绕“吃住行、游购娱、健体闲”，发展多种业态的旅游服务业，提高产 业配套能力。

　　办好旅游季活动。降低景区票价，推出一批精品旅游线路，精心组织好“水上看盘锦”、“空中看盘锦”等系列主题活动。发展壮大旅行社，增加旅游专列批次，启动旅游包机、旅游直通车，加大引客入盘力度，让游客畅游在盘锦、消费在盘锦。

　　充分利用各级各类媒体，组织企业、社会力量共同扩大宣传，将盘锦旅游推向全国、推向世界。

　　（七）支持小微企业加快发展。开展全民创业创新，加快小微企业发展，是促进全市经济社会健康发展的重要举措。我们将进一步优化政策环境，让每个创业者都能实现自己的梦想，形成小企业铺天盖地、大企业顶天立地的生动局面。

　　加强政策扶持和宣传引导。召开促进小微企业发展大会，实施壮大楼宇经济、培育小巨人企业专项行动计划，出台更加有力的扶持政策，激发全社会创业 热情。以大洼总部生态城、兴隆台中央商务区等为载体，加快培育亿元楼宇。加大创业宣传、创业辅导工作力度，厚植创业创新文化，使创业创新成为人们普遍的生 活追求。

　　启动新一轮人才支撑计划，大力引进培养创业创新型人才和转型发展所需的各类人才，构建一支高素质的人才队伍，为全民创业、大众创新提供有力支持。

　　加大制度创新力度。进一步削平创业门槛，放开市场准入，加强市场监管，创造更加公平的竞争环境，用政府权力的“减法”，换取创业投资的“加法”。

　　加大融资服务力度。继续完善小微企业融资担保体系，强化担保支持和政策贴息，积极开展助保贷等业务，千方百计破解小微企业融资难题。

　　加大创业平台建设力度。抓好孵化基地、创业园等服务平台建设，推动各类创业基地向乡镇、街道拓展，为全民创业创新提供全方位的优质服务。

　　（八）积极推进城乡一体化发展。以宜居乡村建设为牵动推进城乡一体化，是造福全市人民的战略工程，也是一项重要的历史任务。我们要保持历史耐心，坚持以人为本，把着力点放到优化城乡空间布局、促进公共服务均等化上来，努力实现城乡一体化建设、一体化管理、一体化发展。

　　科学编制和实施宜居乡村发展规划纲要、镇村建设控详规划，在基础设施、公共服务、商业服务、产业发展四个方面持续发力，引领宜居乡村建设沿着新型城镇化、城乡一体化的正确轨道加速前进。

　　加强农村基础设施建设。村内主要道路实现黑色路面全覆盖。推进农村供水经营主体的公益性回归，提高服务水平。解决4万人农村饮水安全问题。推进 大伙房水库输水配套工程向农村拓展。建成城乡一体的垃圾收运体系，垃圾无害化处理率达到100%。健全落实常态化管理机制。建设50个风貌各异的美丽村。

　　完善农村公共服务。农村燃气普及率达到100%。标准化连锁超市实现行政村全覆盖。镇村公办医疗机构实现统一管理。努力在三年内实现全域客运公交一体化运营。推动教育、卫生、文化等公共服务向农村延伸。

　　加快完善镇域服务业体系，发展都市农业、休闲农业、观光农业、特色旅游等富民产业，因地制宜发展轻工业，以产业发展支撑宜居乡村建设。

　　实施辽河口发展攻坚计划，促进区域均衡发展。大力推进诚通纸制品项目，加快苇纸一体化步伐。发展特色经济，增强造血功能，把石新镇建设成为休闲 观光镇，带动群众走上致富道路。加大政策支持和扶贫力度，抓好生态移民、棚户区改造、社会保障、低保救助等工作，改善困难群众生活，实现精准脱贫。

　　积极推进行政区划调整，形成辽河油田、盘锦港双核支撑，母城、新区双城发展的城市格局。继续支持盘山新县城建设和双台子老城区改造。加快滨海公路绕行线、辽河生态示范路二期等11项重大交通工程建设。完善城市道路、供水、供气、供热、供电、排水等基础设施建设。

　　抓好以信息化为核心的智慧城市建设。拓展市县镇三级政务信息网络功能，整合信息化资源，启动建设云计算中心。加强农村信息基础设施建设，提高信 息化服务水平。完成地下管网普查，建成地下管网智能管理平台。完善地理信息平台。拓展市民卡应用范围。城乡4G网络覆盖率达到98%以上，提升无线宽带接 入率，建设“宽带中国”示范城市。

　　提高城市管理水平。结合全国文明城市创建活动，加大市容市貌、市场秩序等综合整治。加强乡镇街道、村屯社区等基层组织的规范化管理，促进职能转变，提高便民利民服务水平。

　　保护生态环境。深入贯彻落实新环保法。全面实施《盘锦生态市建设规划》。抓好城乡绿化，加强湿地保护。推进蓝天工程，落实大气污染防治措施，最大限度降低雾霾发生，全年达到国家二级标准的天数不低于65%。继续实施碧水工程，持续改善全市主要河流的干流、支流水质。

　　（九）加强以民生改善为重点的社会建设。始终把保障民生作为政府一切工作的出发点和落脚点，坚持尽力而为、量力而行，更加有力地守住底线、突出重点、完善制度、引导舆论，扎实办好60件民生实事，让发展成果更多更公平地惠及人民群众。

　　实施更加积极的就业政策，以创业带动就业。城镇登记失业率控制在3%以内，零就业家庭保持动态为零。对困难家庭高校毕业生实行公益性岗位兜底安置。

　　完善城乡社保体系。提高企业退休人员、城乡居民基础养老金标准和城镇居民医保、新农合补助标准。实施社保市级统筹，加强社保监管。完成两区和辽东湾新区城乡居民医保并轨工作，开展新农合和大病保险“一站式”报销服务。启动省内异地居住人员就医直接结算工作。

　　加大对困难群体救助力度。提高居民低保、农村五保对象供养标准。实施基本医疗救助和重特大疾病救助。扩大低收入老人高龄津贴发放范围，健全完善社会养老服务体系。对700户贫困残疾人家庭实施无障碍改造。

　　推进保障性安居工程建设。改造城市棚户区32807户、农垦职工危房19798户。华润热电一期受益居民扩大到85500户。实施70万平方米“暖房子”改造工程。

　　优先发展教育事业。深化教育综合改革，促进各级各类教育协调均衡发展。推进素质教育，减轻中小学生课业负担。完善扶贫助学体系，不让一个贫困家庭的孩子因贫失学。抓好盘锦职业技术学院省高职示范校建设。

　　积极发展卫生计生事业。推进县级公立医院改革，改扩建镇卫生院10所。提高盛京医院辽东湾分院运营水平。实现镇村基本药物制度全覆盖。实施出生缺陷干预工程，开展农村妇女“两癌”免费检查及救助活动。免费为1000名贫困白内障患者实施复明手术。

　　加快发展文化事业。建成图书馆、青少年活动中心、大剧院、科技馆等文化场馆。开展送戏下乡、送电影进村和优秀传统文化进社区活动。推进农村文化广场建设，实现社区书屋全覆盖。

　　认真筹备第十三届省运会，精心举办红海滩国际马拉松、红海滩自行车骑行、中超联赛等体育赛事，办好第七届市运会。广泛开展全民健身活动，加强全民健身体育设施建设。

　　全力维护社会稳定。坚持不懈开展安全稳定风险定期评估。加大各类隐患排查整治力度，确保安全生产形势持续好转。建立食品药品全程监督追溯信息化 平台。扩大优质食材配送范围。加强社会安全管理，完善社会治安防控、全民反恐工作体系，严厉打击各类违法犯罪行为，规范犬类管理，整合视频监控系统，进一 步增强群众安全感。把信访纳入法治化轨道，健全维护群众利益的制度体系，有效化解社会矛盾。

　　积极支持驻盘部队、国防教育和后备力量建设。深入开展双拥共建工作，夺取全国双拥模范城“五连冠”。实施少数民族发展惠民工程，加强宗教事务管理，促进民族关系更加和睦、宗教关系更加和谐。

　　科学编制“十三五”规划。抓好防震减灾、人民防空、妇女儿童、老龄慈善、地方志、统计、物价、质监、档案、外事等各项工作。

　　（十）推进法治政府建设。全面落实依法治市实施意见，建设职能科学、权责法定、执法严明、公开公正、廉洁高效、守法诚信的法治政府，提高政府治理现代化水平。

　　依法全面履行政府职能。着力推进机构、职能、程序、责任法定化，切实做到法无授权不可为、法定职责必须为。

　　推进政府运行机制法治化。坚持用法治思维和法治方式开展工作，确保政府工作在法治轨道上有序运行。健全依法决策机制，强化实施政府法律顾问制度，严格执行重大决策合法性审查机制。深化行政执法体制改革，严格规范执法行为。

　　创新政府服务管理方式。深入推进政务公开，让权力在阳光下运行。优化完善政府绩效考核及相关制度，全面实现政府工作的规范化、法治化。

　　强化对行政权力的制约和监督。自觉接受市人大及其常委会的法律监督、工作监督和市政协民主监督，健全与民主党派、工商联和无党派人士的联系制 度，认真办理人大代表议案、建议和政协提案。加大政务督查、行政监察、审计监督力度。高度重视司法、舆论、社会监督，使政府工作全面接受群众监督，充分体 现人民意愿。

　　各位代表，当前，盘锦正处在转型发展的攻坚期、爬坡过坎的关键期。面对新形势、新任务，我们要进一步加强政府自身建设，创造无愧于时代、无愧于人民的新业绩。

　　我们要深化“四风”整治，巩固党的群众路线教育实践活动成果，推动作风建设规范化、常态化和制度化。

　　我们要敢于担当，敢于负责，敢于攻坚，确保今年各项任务落到实处。

　　我们要说实话、办实事、求实效，做到言必信、行必果，切实增强政府公信力。

　　我们要加大惩治和预防腐败力度，严肃查处各类违纪违法案件，营造风清气正的政治生态和从政环境。

　　各位代表，盘锦已经站在新的历史起点上，转型发展的步伐更加坚实有力。让我们在市委的坚强领导下，紧紧依靠全市人民，真抓实干，努力拼搏，为全面建成小康社会，开启向海发展全面转型以港强市新航程而努力奋斗！
